# Sigma Receptor (σR) Ligands with Antiproliferative and Anticancer Activity

**DOI:** 10.3390/molecules22091408

**Published:** 2017-08-25

**Authors:** Markos-Orestis Georgiadis, Olga Karoutzou, Angeliki-Sofia Foscolos, Ioannis Papanastasiou

**Affiliations:** School of Health Sciences, Department of Pharmacy, Division of Pharmaceutical Chemistry, National and Kapodistrian University of Athens, Panepistimioupoli-Zografou, 15784 Athens, Greece; aopf_9@hotmail.com (M.-O.G.); olgakarou@gmail.com (O.K.); angelique.lumiere@windowslive.com (A.-S.F.)

**Keywords:** sigma 1 receptor antagonist, sigma 2 receptor agonist, antiproliferative activity, anticancer activity, radiolabeled and fluorescent probes, biomarkers

## Abstract

Sigma receptor (σR) ligands have proven to be useful as cancer diagnostics and anticancer therapeutics and their ligands have been developed as molecular probes in oncology. Moreover, various σR ligands generate cancer cell death in vitro and in vivo. These σR ligands have exhibited promising results against numerous human and rodent cancers and are investigated under preclinical and clinical study trials, indicating a new category of drugs in cancer therapy.

## 1. Introduction

Sigma receptors (σRs) have been recently referred in cancer pathophysiology. Initially, they were identified as opiate receptors and their description was based on the pharmacological evaluation of (±)-SKF-10,047 (*N*-allylnormetazocine), morphine and ketazocine in the chronic spinal dog model [1]. Three types of the opiate receptors were suggested and named by the corresponding greek symbol: μ for morphine, κ for ketazocine, and σ for (±)-SKF-10,047 [2,3]. Nevertheless, σRs have been classified as a distinct pharmacological receptor class and are unrelated to opioid receptors [1,4,5]. They consist of a ubiquitously expressed different binding site in the CNS and other peripheral tissues [6,7,8,9]. No endogenous ligand was known until the characterization of dimethyltryptamine (DMT) [10,11]. Steroid hormones (particularly progesterone) and sphingolipid-derived amines might also be included as endogenous ligands [12].

Originally, two types of sigma receptors (σRs) were identified, sigma 1 receptor (σ1R), which was first cloned in 1996, and sigma 2 receptor (σ2R), which has not been cloned yet [6,13,14,15,16,17]. One more type has been suggested, sigma 3 (σ3R), but it has not been defined adequately [18]. σ1R and σ2R have recently been involved in apoptosis (programmed cell death) [4,19,20,21,22,23]. σ1R and σ2R are highly expressed in cancer cells and up-regulated prior to mitosis [24,25], suggesting important cellular functions in cancer. σ1R antagonists [26,27,28] deactivate the receptor activity, which is anti-apoptotic and neuroprotective [4,16,19,29,30] and σ2R agonists [20,21,22] stimulate the receptor activity and sensitizes cancer cells for apoptosis [21,22,31]. Although there is considerable evidence of antiproliferative and cytotoxic activity for σ1R antagonists, σ2R agonists and mixed σ1R/σ2R ligands [20,21,22,23,26], the mechanism of action is still elusive.

Both σR types are overexpressed in numerous human cancer tissues, such as small- and non-small-cell lung carcinoma [24,32], large-cell carcinoma (NCI-H1299 and NCI-H838) [33], renal carcinoma [24], colon carcinoma (HCT-15 and HCT-16) [34], sarcoma [33], brain tumors (CNS U51) [35], breast cancer (MCF-7. T47D and SKBr3) and breast ductal carcinoma (T47D) [32], melanoma (A375) [24], glioblastoma [24], adenocarcinoma (line 66), neuroblastoma (BE(2) and SK-N-SH) [24], prostate cancer (DU-145, PC3 and LnCap) [24], pancreas (MiaPaca2 and BX-PC3), liver (SKHep1), ovarian carcinoma (ICROV-1 and OVCAR-5) and leukemia (HL-60) [24]. Consequently, many pharmaceutical agents acting at the σRs have been used in the treatment of cancer and are receiving considerable attention.

A functional assay to define the agonist/antagonist behavior of σR ligands does not exist at the time of writing this review. Many σR ligands with various scaffolds have been evaluated as cytotoxic in a variety of cancer cell lines by activating caspase-dependent and caspase-independent apoptosis. This deviation in the mechanism of action can be used to define σR ligands as agonists or antagonists. More specifically, ligands that induce caspase-3 activation and cytotoxicity are commonly accepted as σR agonists, whereas compounds that do not cause caspase-3 activation and cytotoxicity are considered as antagonists [36,37].

## 2. Sigma Receptors (σRs)

σ1R is a polypeptide of molecular weight (MW) 29 kDa that comprises 223 amino acids and is not similar to known receptors, except for a 66.4% homology with a yeast C8-C7 sterol isomerase [6,7,9,38,39,40,41,42]. The σ1Rs are expressed in various tissues, and especially in the cardiac tissue and the spleen. They are widely located in the endoplasmic reticulum and the plasma membrane [6]. They are important for the modulation of cation channels (K^+^, Na^+^ and Ca^2+^). σ1Rs are intracellular receptors that can translocate inside cells and act as chaperone proteins [43,44]. Chaperone proteins are responsible for the correct folding of other proteins, during their synthesis or function [45]. σ1Rs regulate Ca^2+^ signaling via the inositol triphosphate [IP3] receptor and, in particular, they ensure the Ca^2+^ signaling from endoplasmic reticulum (ER) into mitochondrion. Under cell stress conditions, the Ca^2+^ homeostasis in the ER is perturbed resulting in resistance to the potential apoptosis. Moreover, σ1Rs modulate K^+^ channels in pituitary and brain cells through G protein coupling or protein-protein interactions [46]. The cell shrinkage, which is necessary for programmed cell death (apoptosis) [47,48,49], is mediated through K^+^ loss. Moreover, σ1R is assumed to be involved in tumor genesis, as the corresponding receptor gene is a target of the oncogene c-Myc [50]. It has been shown that σ1R antagonists induce caspase-dependent apoptosis [26,51], whereas σ1R agonists prevent caspase activation [4,52]. For this reason, σ1R antagonists have antiproliferative and cytotoxic activity and the σ1R agonists are anti-apoptotic and neuroprotective [16,53].

The σ2 protein was initially characterized as the progesterone receptor membrane component 1 (PGRMC1) [54]. Even if this receptor has not been cloned yet, the corresponding gene is presumed to encode a protein of MW 21.5 kDa. In contrast to σ1Rs that dynamically translocate, σ2Rs are located in the lipid raft and are coupled with the PGRMC1 complex, EGFR, mTOR, caspases, and various ion channels [55]. σ2Rs appear to interfere in cell cycle and apoptosis by regulating the sphingolipid pathway. In particular, they produce an increase in ceramide, a sphingolipid second messenger in cell proliferation [56]. Moreover, their activation leads to high intracellular calcium concentrations that can in turn activate proteases, nucleases and other enzymes that mediate apoptosis. The σ2R is overexpressed in many tumor cell lines, thus it constitutes an attractive target for cancer diagnosis and treatment. σ2R could be used as biomarker of the tumor proliferative status, due to its high density in the proliferating tumor cells [57]. Thus, σ2R ligands could be useful for imaging cancer in vivo, using techniques such as positron emission tomography (PET) [58] or single-photon emission computerized tomography (SPECT) [59,60].

σ2R agonists and antagonists produce different effects. σ2R agonists have antiproliferative and cytotoxic activity in tumor cells in vitro as well as in vivo [61]. They provoke cell death via a multitude of distinct pathways such as caspase-dependent and -independent mechanisms [62], generation of reactive oxygen species (ROS) and autophagy [21,63]. More specifically, the caspase-dependent mechanism triggers caspase 3, 8 and 9. Another potentially exploitable fact is the interaction of σ2R ligands with p-glycoprotein (P-gp) efflux pump and their ability to decrease P-gp levels [64,65]. Nearly half of human cancers develop resistance to antineoplastic therapy due to overexpression of P-gp. Therefore, σ2R agonists can act as single antitumor agents without resistance problems or can be co-administrated with classic antineoplastic medication to reduce the Multi Drug Resistance (MDR) effect.

## 3. Structure Affinity Relationship of Sigma Receptors Modulators

σRs have historically invoked scientific interest due to their accommodation of different structural ligands. Consequently, a great variety of drug classes can bind to them with high affinity [66,67]. This broad structural diversity among σR ligands can be explained via a multitude of hypothesis, the most prevalent of whom suggests that the receptors possess dynamic structures, sufficiently flexible to accommodate all these structurally diverse compounds [68]. In this case, a single pharmacophore model that defines a specific three-dimensional space for pharmacophore groups may be difficult or even impossible to exist. Nevertheless, numerous two-dimensional pictorial pharmacophore models have been proposed for σR ligands.

### 3.1. σ-1 Selective Ligands

#### 3.1.1. Gilligan Model

**Figure 1:**
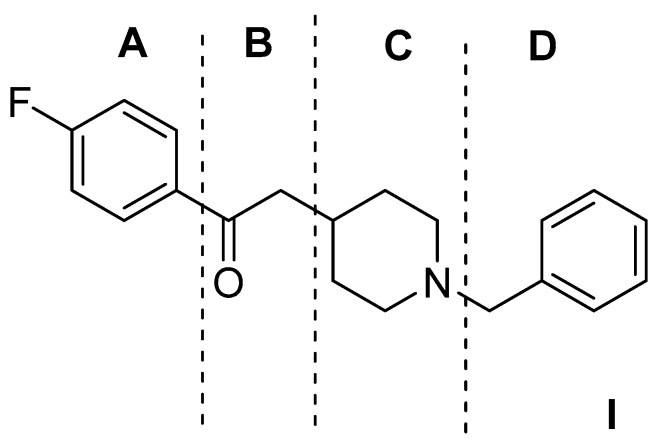
Gilligan model: (1) a distal aromatic ring (Region A); (2) a nitrogen heterocycle (Region C); (3) a space between the heterocycle and the distal aromatic ring (Region B); and (4) a substituent on the nitrogen heterocycle (Region D).

Gilligan et al. [69] identified a lead compound selective for σR (Ki = 6 nM). The lead compound **I** was analyzed into four sections, corresponding to four pharmacophore moieties, as depicted in Figure 1.

#### 3.1.2. Glennon/Ablordeppey Model

**Figure 2:**
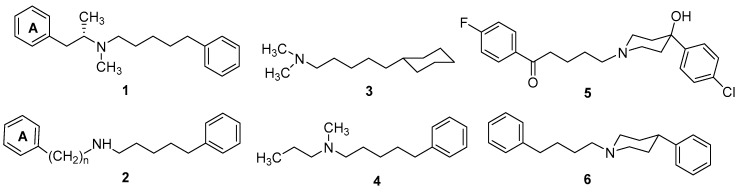
Structural modifications related to σ1R binding affinity.

This model is derived from studies that aimed at identifying a pharmacophore for the binding of benzomorphan analogs at σRs. It became immediately obvious that an intact benzomorphan moiety was not required for high-affinity binding. Compound **1** was shown to possess high affinity for σRs. Appropriate aryl substituents in the phenylethylamine portion of the molecule (including fused-ring structures) or decrease of the length of side chain by one or two methylene groups reserve high affinity (σKi < 10 nM) [70]. Both secondary and tertiary amines are potent ligands; however, one of the tertiary amine substituents could not be much larger than a methyl group [71]. Moreover, the phenylpentyl moiety, not a phenylethyl moiety, of **1**-type compounds was crucial for binding at σRs. The comparison of several phenylpentylamines **2** where (CH_2_)*_n_* was varied from *n* = 1 to *n* = 4 (σKi = 2.0–2.7 nM) showed that variation of Phenyl-A to *N* chainlength had no significant impact on affinity [69,72] (Figure 2).

**Figure 3:**
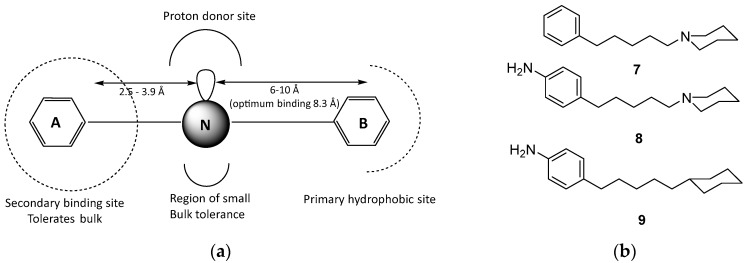
(**a**) Initial Glennon/Ablordeppey pharmacophore model [78]; and (**b**) structural modifications of the basic nitrogen atom.

On the other hand, either or both of the aromatic rings could be replaced by a cyclohexyl ring proving that the interaction with σRs involves a hydrophobic rather than an aromatic-type or π–π stacking interaction. Moreover, Phenyl-A could be deleted without impact on affinity; for example, derivatives **3** (σKi = 2.6 nM) and **4** (σKi = 2.4 nM) remain as potent as compound **2** [73,74]. A phenylpiperidine or phenylpiperazine ring has almost the same dimensions with a phenylethylamine and it was proven that such derivatives are also potent [75]. It was reasoned that, if the phenylpentylamine moiety is a significant pharmacophore contributor, it should be possible to extend the butyrophenone chain of haloperidol to valerophenone. Indeed, valerophenone **5** (σKi = 2.3 nM) was found to have several-fold higher affinity than haloperidol (CTKi = 10 nM). Removal of polar substituents in the phenyl ring, to afford phenylpentylamine **6**, resulted in increase of affinity (**6**; CTKi = 0.9 nM) [76]. At the time, compound **6** exhibited the highest σR affinity. The next set of experiments examined the impact of the *N*-alkyl substitution. As long as one of the *N*-alkyl substituents is a methyl group, the nature of the second substituent has limited impact on affinity, provided it is at least three carbon atoms in length. This evidence supported the hypothesis of a hydrophobic binding pocket of limited size, and that, as long as this hydrophobic binding requirement was met, derivatives presented high affinity. Further bulk substituent was probably accommodated in an associated region of bulk tolerance, and did not usually increase affinity [77] (Figure 2). All the above results were used by Glennon and Ablodeppey to propose an initial pharmacophore model for high affinity σR ligands, which is depicted in Figure 3.

Another question was the role of the basic nitrogen atom. Several studies had presented that σR ligands did not require a basic amine. Steroids, for instance, are σR ligands [79]. An amino group was shown to be well tolerated in the phenyl ring of derivative **7** (**8**, σKi = 38 nM). Subsequently, the piperidine amino group was deleted, giving compound **9**, which (σKi > 36,000 nM) was >50,000-fold less potent than adduct **7**. The presence and location of the basic amine proven to be important for binding [79,80]. Various and diverse compounds have been demonstrated to be σR ligands. However, two major features have been revealed: (1) many bind with affinity only in the micromolar or very high nanomolar range; and (2) most display an aryl or hydrophobic ring separated from a basic tertiary amine by four to seven atoms. Although a five-atom linker seems optimal, compounds with longer alkyl chains might simply interact with a hydrophobic binding site on the receptor in a less efficient manner than phenyl or cyclohexyl groups. Compounds with longer chains might also fold back somewhat to be accommodated by the receptor. In any case, long chains are well tolerated.

### 3.2. σ-2 Selective Ligands

The synthesis of selective ligands for the σ2R versus the σ1R has always been a challenge. The fact that σ2R accommodates very different structures has made it difficult to produce a pharmacophore model for rational design of σ2R ligands [61,81].

#### 3.2.1. Conformationally Restricted Amine Derivatives

**Figure 4:**
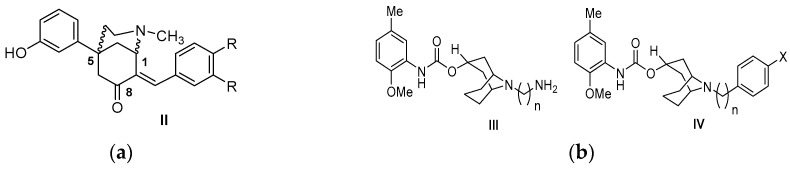
Conformationally restricted amine selective σ2R derivatives: (**a**) Benzomorphan-7-one analogs; and (**b**) Granatane analogs.

The first class of σ2R selective ligands was the benzomorphan-7-one analogs, as illustrated in Figure 4 [82].

The most selective σ2R ligands were (+)-1*R*,5*R*-(*E*)-8-benzylidene-5-(3-hydroxyphenyl)-2-methyl-morphan-7-one (**CB-64D**, σ2Ki = 16.5 nM, σ1/σ2Ki = 185) and (+)-1*R*,5*R*-(*E*)-8-(3,4-dichlorobenzylidene)-5-(3-hydroxyphenyl)-2-methylmorphan-7-one (**CB-184**, σ2Ki = 13.4 nM, σ1/σ2Ki = 555). These benzomorphans displayed affinity for the μ opioid and σ2 receptors, because the aforementioned receptors share the same enantioselectivity. The (+)-isomers are selective for the σ2R, while the (−)-isomers have a higher binding affinity for the σ1R. This chemical category of derivatives is merged with granatane- or tropane-related bicyclo-analogs. The 9-*N* atom of the granatane ring can accommodate bulky substitutions without a significant loss of σ2R affinity and selectivity. A *N*-substitution with an additional nitrogen atom that is four or more carbon atoms apart enhances σ2R binding affinity. A *N*-aromatic substitution can also be accommodated, but is not crucial for σ2R affinity or selectivity [83,84,85].

#### 3.2.2. Siramesine-Related Indole Analogs

**Figure 5:**
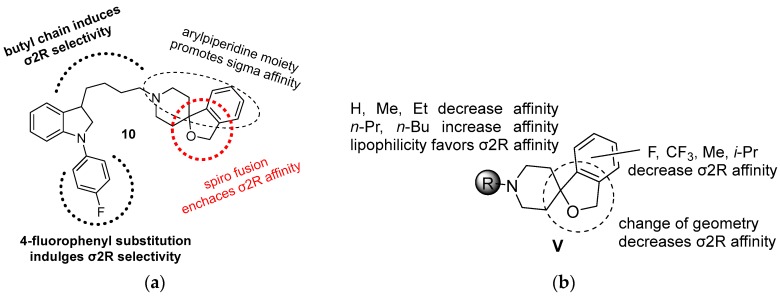
(**a**) Siramesine or Lu 28-179; and (**b**) structural modifications of siramesine analogs.

**Siramesine** (Lu 28-179) was designed as a low-efficacy serotonin 5-HT1A agonist for treating depression and anxiety disorders [86], but it was later revealed that **siramesine** displayed a subnanomolar affinity for σ2R and a 140-fold selectivity for σ2R versus σ1R. This remark led to the development of a new series of siramesine analogs (σ2Ki = 0.12 nM, σ1/σ2Ki = 140) (Figure 5) [86,87]. *N-s*mall alkyl substitution decrease sigma affinity, while *n*-propyl, *n*-butyl groups lead to an increase of sigma binding affinity with a corresponding shift towards σ2R selectivity. The introduction of a fluorine atom or a trifluoromethyl group at the spiropiperidine benzene ring reduces σ2R affinity or selectivity. In addition, when the geometry of spiro-system changes, the affinity and selectivity towards σ2R decrease [86,87] (Figure 5).

#### 3.2.3. Conformationally Flexible Amine Derivatives

**Figure 6:**
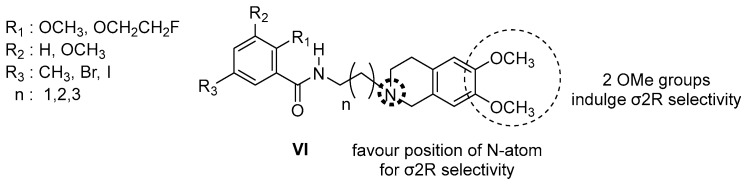
Conformationally flexible benzamide analogs.

Benzamide highly selective σ2R derivatives are illustrated in Figure 6. These compounds were initially designed as dopamine D3 selective antagonists and partial agonists, but the structural modifications to improve the “drug-like” properties generated the aforementioned σ2R selective ligands [88,89]. The dimethoxy groups of the 6,7-dimethoxytetrahydroisoquinolines are important for maintaining a high affinity for the σ2R binding [89]. A restricted amine structure is beneficial for σ2R binding [90]. The aromatic substitution of the benzamide can tolerate large alkyl chains and an intramolecular hydrogen bond may be formed between the oxygen of the ortho-methoxy group (vide R_1_, Figure 6) on the benzamide and the amide NH. This bond could be important for σ2R binding [65,91,92].

**Figure 7:**
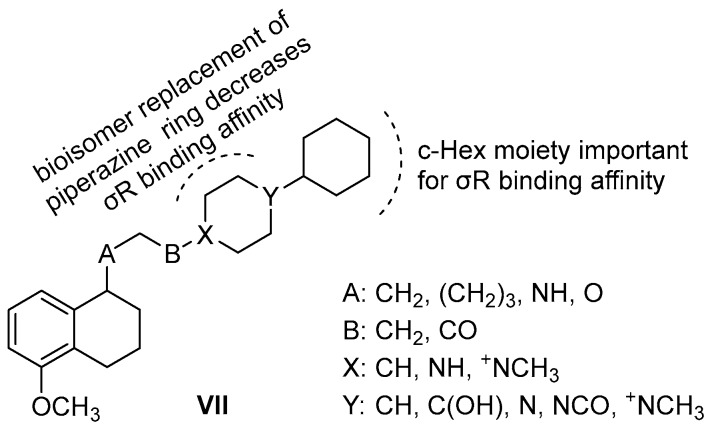
Cyclohexylpiperazines and cyclohexylpiperdines analogs.

Cyclohexylpiperazines and cyclohexylpiperdines have been studied for both sigma receptors, since these compounds are highly potent and nonselective σ1/2R ligands (Figure 7). The Structure–Activity Relationship of this category of compounds supported the hypothesis that the lipophilicity is correlated to the antiproliferative activity mediated by the σ2R [93]. The higher lipophilicity indulges higher affinity and efficacy.

In the above-mentioned model in Figure 7, *N*-cyclohexylpiperazine moiety proves to be an optimal substituent of this category of derivatives. Quaternary amines are also capable of binding to σ2R with moderate affinity and selectivity over σ1R. When a carbazole moiety replaced the 5-methoxytetraline resulted a significant decrease in σ1R binding affinity and a 273-fold selectivity for σ2R [93,94].

## 4. σ-Receptor (σR) Ligands in Cancer Research

σR are expressed in large quantities in the majority of cancer cell lines, suggesting that σR ligands can be used as potential tools in the treatment or diagnosis of various types of cancer [12,35,94,95].

As far as diagnosis is concerned, σR ligands can be used for diagnostic imaging using PET or SPECT. Their use as diagnostic tools is based on the aforementioned overexpression of σR in different types of cancer and as on the ability of σRs to internalize their ligands, as well. Moreover, several σR ligands contain an iodine or fluoride atom in their chemical structure, which can be easily substituted with the corresponding radioisotope [59,96,97,98,99]. Several preclinical studies evaluated the potential use of radiolabeled sigma-ligands as imaging agents in melanoma [100], breast cancer [101,102], prostate cancer [101], and lung cancer [100]. Everaert et al. highlighted that malignant melanomas can be detected in patients with 87% accuracy and 64% sensitivity at the lesion site, using a radiolabeled benzamide σ1R ligand **^123^I-IDAB** (^123^I-*N*-(2-diethylaminoethyl)-4-iodobenzamide) [100,102]. A preliminary clinical study showed that the σ1R ligand **^123^I-IMBA** (^123^I-*N*-[2-(1′-piperidinyl)-ethyl]-3-iodo-4-methoxybenzamide), is accumulated in most breast tumors in vivo due to uptake by or a high density of σRs in cancer cells, in comparison to normal tissue [102].

**Figure 8:**
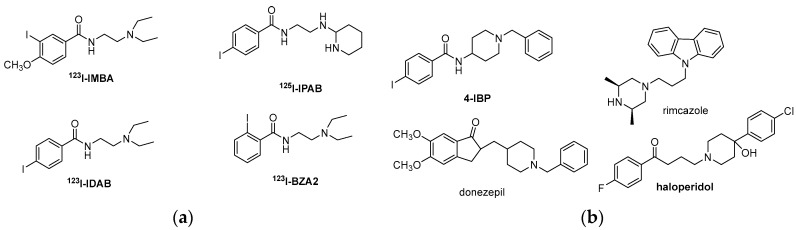
(**a**) Radiolabeled selective σR1 ligands that have been used in pre-clinical studies of tumor imaging; and (**b**) σR1 ligands that are being used in cancer treatment.

Leaf Huang et al. have been using selective σ1R ligands for delivering drugs to human cancer cells. Their group designed the benzamide derivative **^125^I-IPAB** (^125^I-(2-piperidinylaminoethyl)-4-iodobenzamide) [103,104] and incorporated it into liposomes containing doxorubicin to specifically deliver the drug to a prostate cancer cell line (DU-145) [105]. The benzamide-conjugated liposomal doxorubicin exhibited significantly higher antiproliferative activity against DU-145 cells than against non-targeted liposomal doxorubicin in vitro, and better accumulation within the tumor in vivo in a xenograft animal tumor model. Moreover, intravenous administration of the targeted liposomal doxorubicin displayed significant growth inhibition of established DU-145 tumors in nude mice, while simultaneously reducing the drug toxicity [105]. This technique was later followed by nanoparticles containing the σR ligand **^123^I-IDAB** to target the delivery of antisense oligodeoxynucleotide and siRNA to lung cancer cells in vitro and in vivo, as well as to the B16F10 mouse melanoma lung metastasis model [106,107]. A phase II clinical trial proved that **^123^I-BZA** (^123^I-*N*-(2-diethylaminoethyl)-4-iodobenzamide) was useful as scintigram in diagnosis of ocular melanoma [108]. Another isomer benzamide adduct of this series of radiolabeled derivatives, which is used in the identification of melanoma metastases is **^123^I-BZA2** (^123^I-*N*-(2-diethylaminoethyl)-2-iodobenzamide). **^123^I-BZA2** was studied in a multicenter Phase III clinical trial and might lead to a new treatment strategy of metastatic melanoma patients harboring melanin-positive metastases [109] (Figure 8).

Recently, various σ1R ligands have been examined for cancer chemotherapy either in conjunction with other anticancer treatments or as monotherapy. It was first shown that σ1R ligand **4-IBP** (4-(*N*-benzylpiperidin-4-yl)-4-iodobenzamide), increased the antitumor effects of temozolomide and irinotecan in vivo, a process that appears to involve the Rho guanine nucleotide dissociation inhibitor (RhoGDI) and glucosyl-ceramide synthase (GCS) [110]. It has also been demonstrated that various σ1R ligands, including current antipsychotic drugs, display antiproliferative activity with mitotic arrest in highly diffusive and migrant glioblastoma (GBM) cells in vitro. Moreover, it was observed that donepezil could provide the same additive benefit to temozolomide treatment as **4-IBP** in vivo [111]. **Rimcazole**, a σ1R ligand for the treatment of schizophrenia, was recently found to kill selectively tumor cells by a process involving HIF-1a, and has now been re-profiled for cancer chemotherapy. **Donezepil**, another σ1R ligand for the treatment of Alzheimer’s disease, is being used in chemotherapy for small cell lung cancer and as adjunctive therapy in brain tumors [112]. **Haloperidol**, known antipsychotic drug and σ1R antagonist, promotes ferroptosis in hepatocellular carcinoma cells [113].

**Figure 9:**
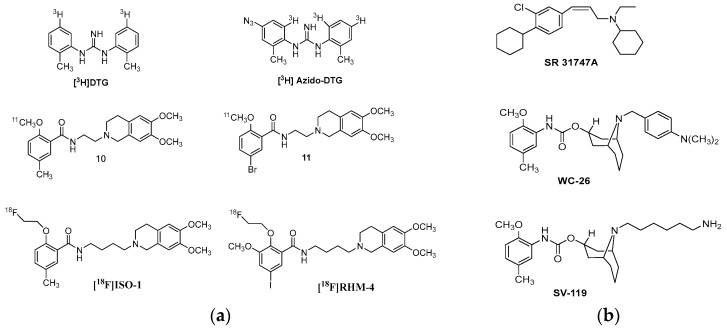
(**a**) Radiolabeled σ2R ligands for in vitro binding studies. ^11^C-Labeled and ^18^F-labeled conformational flexible benzamide analogs; (**b**) σ2R ligands for in vivo binding studies.

σ2R ligands have been used for more than 20 years as radiolabeled and fluorescent probes to provide structural information of the correspondent receptor and highlight solid tumors as biomarkers [37,114,115,116]. **[^3^H]DTG** is one of the most known radioligand in the study of σ2R [114]. **[^3^H]azido-DTG** was an important analog in the characterization of the molecular weight of σ1R and σ2R [66,114]. Various conformationally flexible benzamides (e.g., vide Figure 9, compounds **10** and **11**) are selective radiotracers for imaging the proliferative status of tumors in vivo with PET [85,115,116]. The 2-methoxy group of the previous derivatives facilitates the preparation of ^11^C-labeladed derivatives due to alkylation of the corresponding 2-hydroxy precursors. In the next generation of radiotracers, the 2-methoxy group was replaced by the 2-fluoroethoxy moiety, because the ^18^F-labeled tracers allow imaging studies to be conducted in due course after the radiotracer injection [117]. MicroPET imaging studies use **[^18^F]ISO-1** in a rodent model of breast cancer and **[^18^F]RHM-4** in a rat model of brain cancer [118]. The former is in clinical Phase I study for imaging solid tumors with PET. Different clinical trials are completed or still going on in various cancer types (primary and metastatic breast cancer, head and neck cancer, and diffuse large B-cell lymphoma) [119].

Several reports describe the antiproliferative and anticancer activity of σ2R ligands in variable cell lines and tumors [32,120,121] (Table 1). The last years, it has become obvious that σ2R ligands have significant role in anticancer therapy, as they provoke cancer cell death [32,121]. **Siramesine** was active against all cancer cell lines tested. Recent experiments of **SV-119** and its congeners have been conducted in a pancreas tumor model with significant results, even though the mechanism of reaction is still elusive [122,123]. **SR31747A**, a mixed σ1/2R and human sterol isomerase binding affinity, has been tested for its anticancer activity, due to its efficacy at σRs. The significant in vitro pharmacological evaluation made **SR31747A** enter clinical trials for the treatment of androgen prostate cancer [124].

Non-selective σR ligands, described as mixed σ1/2R ligands, are used in cancer diagnosis and therapy. Even though σ1R and σ2R are structurally different proteins, σ1R has been characterized as a chaperone protein [43,44] and σ2R seems to belong to a progesterone receptor complex (PGRMC1) [54], both of them share common ligands. Various publications refer to σR ligands that do not present receptor selectivity [31,125]. Cyclohexylpiperazine adducts have already been reported in the current context as non-selective σR ligands. Benzylpiperazines with σ1/2R affinity exhibit high antiproliferative activity against a wide panel of cancer cell lines [31]. Recent publications presented 1,4-benzodioxane- and 1,3-dioxolane-coupled benzylpiperazines as mixed σ1/2R ligands [126]. Moreover, the defect in binding selectivity becomes an advantage in tumor signaling. The overexpression of both σ1R and σ2R in prostate tumor and neuroblastoma [12,23,99] suggests that a dual σR radioligand might present an enhanced tumor targeting compared to a selective radioligand for a single σR subtype [127]. Another study confirms the same conclusion in radiolabeled pulmonary σR assigment [128].

## 5. Adamantane Derivatives with σR Binding Affinity, Antiproliferative and Anticancer Activity

**Figure 10:**
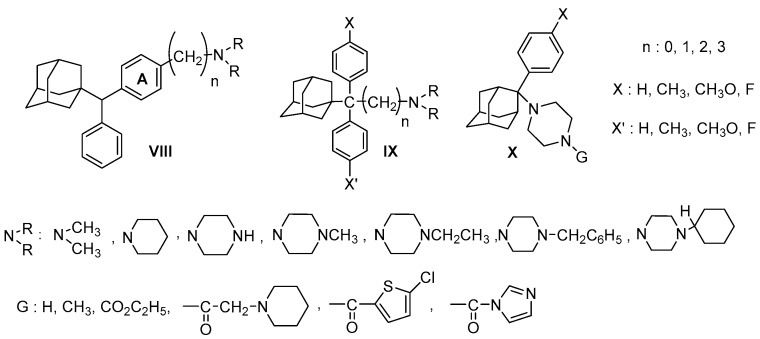
Adamantane derivatives with σR binding addinity and antiproliferative or anticancer activity.

Adamantane skeleton, usually characterized as “lipohilic bullet” [129], is the structural backbone of many drugs in clinical practice [130]. Various adamantane derivatives present σR binding affinity not related to antiproliferative or anticancer activity [125,131,132,133,134,135]. However, the following adamantane phenylalkylamines **VIII**, **IX** and **X**, as illustrated in Figure 10, exhibit σR binding affinity in combination with antiproliferative and anticancer activity [136,137,138,139,140].

The aforementioned adamantane adducts present the structural requirements for σR binding affinity (Figure 10). In the first adamantane scaffold **VIII**, benzene ring A is attached to the first piperazine nitrogen via a chain of three atoms (N, 2C) and in template **IX**, the benzene rings are linked to an amine nitrogen atom via a spacer of one, two and three methylene carbons. The substitution of the adamantane moiety has changed in 1-(2-aryl-2-adamantyl)piperazine derivatives **X**. All the above adamantane derivatives have a significant binding affinity for the σ1R and σ2R at a low nanomolecular range. Their antiproliferative activity against numerous cancer cell lines (colon, prostate, breast, ovarian, central nervous system, leukemia, pancreas, liver) was significant. These results in conjunction with their affinity for site 2 of the Na^+^ channels imply that the adamantane phenylalkylamines **VIII**, **IX** and **X** have the pharmacological profile of mixed σ1/σ2R ligands [136,137,138,139].

**Figure 11:**
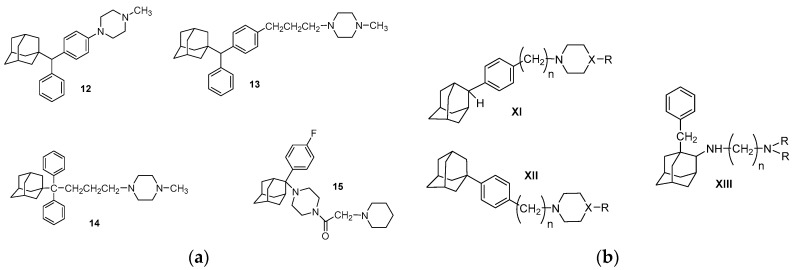
(**a**) Adamantane phenylalkylamines **12**–**15**; and (**b**) adamantane adducts with antiproliferative potency.

1-Methyl-4-{3-[4-[α-(1-adamantyl)phenyl]phenyl]propyl}piperazine (**13**) presented an acceptable toxicological profile associated with an interesting antiangiogenic activity against tumors and was particularly prominent in (BxPC-3) pancreas, (DU-145 and PC3) prostate, (OVCAR-5) ovarian and (HL-60) leukemia xenografts on SCID mice [136]. 1-Methyl-4-{4-[α-(1-adamantyl) phenylmethyl]phenyl}piperazine (**12**) (σ1Ki = 3.2 nM, σ2Ki = 32 nM, σ1/σ2Ki = 11.8) was prominent in pancreas [137]. 4-[4,4-Diphenyl-4-(1-adamantyl)butyl]-1-methylpiperazine (**14**) (σ1Ki = 15 nM, σ2Ki = 60 nM, σ1/σ2Ki = 4) displays selective action against ovarian cancer on mice (IGROV-1) and presented as potent as cisplatin [138]. The above adamantane adducts were also tested with a prototypical study (formaline test) of their effect in putative neuropathic pain induced by anticancer drug Paclitaxel [28,29,30] and proved to be putative analgesic agents. 1-[2-(4-Fluorophenyl)-2-adamantyl]-4-(1-piperidineacetyl)piperazine (**15**) had also notable antitumor activity [139] (Figure 11).

Finally, the following phenylalkylamines analogs with general type **XI** [141], **XII** [142] and **XIII** [143] have been reported for their antiproliferative activity and due to the similarities of their scaffold with compounds **VIII** and **IX**, it can be assumed that they act as σR ligands, although their binding affinities have not been investigated yet (Figure 11).

## 6. Conclusions

The reports described in our current review induce a new category of drugs against cancer. σRs are still poorly understood, but it has become increasingly apparent that these receptors have a significant role in cancer pathophysiology. σ1R Antagonists, σ2R agonists and mixed σ1R/σ2R ligands have antiproliferative and cytotoxic activity, even though their mechanism of action is under investigation. The fact that many σR ligands are in preclinical and clinical phase trials is a testimony of this improvement in cancer therapy.

## Figures and Tables

**Table 1:** σ2R ligands and their targets.

Origin	Species	Cell Line	σ2R Ligand
breast cancer	human	T47 D	**[^3^H]DTG**
	human	MCF7	**[^3^H]DTG**
	mouse	EMT-6	**[^3^H]DTG**,**WC-26**,**SV-119**
colon cancer	human	primary tumor	**[^3^H]DTG**
leukemia	human	Th-P1	**[^3^H]DTG**
lung	human	NCI-H727	**[^3^H]DTG**
melanoma	human	A375	**[^3^H]DTG**
	human	MDA MB-435	**WC-26**,**SV-119**
neurologic	human	U-138MG	**[^3^H]DTG**
	human	primary tumor	**[^3^H]DTG**
	mouse	NB41A3	**[^3^H]DTG**
	mouse	N1E-115	**[^3^H]DTG**
	rat	C6	**[^3^H]DTG**
pancreas cancer	mouse	Panc-02	**SV-119**
	human	Panc-01	**SV-119**
	human	AsPc-1	**SV-119**
	human	CFPAC	**SV-119**
prostate	human	LNCaP	**[^3^H]DTG**
sarcoma	human	primary tumor	**[^3^H]DTG**

## References

[1] Martin W.R., Eades C.G., Thompson J.A., Huppler R.E., Gilbert P.E. (1976). The effects of morphine- and nalorphine-like drugs in the nondependent and morphine-dependent chronic spinal dog. J. Pharmacol. Exp. Ther..

[2] Matsumoto R.R., Matsumoto R.R., Bowen W.D., Su T.-P. (2007). Sigma Receptors: Historical perspective and background. Sigma Receptors: Chemistry, Cell Biology and Clinical Implications.

[3] Collier T.L., Waterhouse R.N., Kassiou M. (2007). Imaging sigma receptors: Applications in drug development. Curr. Pharm. Des..

[4] Su T.-P., Hayashi T. (2003). Understanding the molecular mechanism of sigma-1 receptors: Towards a hypothesis that sigma-1 receptors are intracellular amplifiers for signal transduction. Curr. Med. Chem..

[5] Bowen D.W. (2000). Sigma receptors: Recent advances and new clinical potentials. Pharm. Acta Helv..

[6] Hanner M., Moebius F., Flandorfer A., Knaus H.G., Striessnig J., Kempner E., Glossmann H. (1996). Purification, molecular cloning, and expression of the mammalian sigma 1-binding site. Proc. Natl. Acad. Sci. USA.

[7] Pan Y.X., Mei J., Xu J., Wan B.-L., Zuckerman A., Pasternak G.W. (1998). Cloning and characterization of a mouse sigma1 receptor. J. Neurochem..

[8] Seth P., Fei Y.J., Li H.W., Leibach F.H., Ganapathy V. (1998). Cloning and functional characterization of a sigma receptor from rat brain. J. Neurochem..

[9] Seth P., Leibach F.H., Ganapathy V. (1997). Cloning and structural analysis of the cDNA and the gene encoding the murine type 1 sigma receptor. Biochem. Biophys. Res. Commun..

[10] Fontanilla D., Johannessen M., Hajipour A., Cozzi N.V., Jackson M.B., Ruoho A.E. (2009). The hallucinogen *N,N*-dimethyltryptamine (DMT) is an endogenous sigma-1 receptor regulator. Science.

[11] Su T.P., Hayashi T., Vaupel D.B. (2009). When the endogenous hallucinogenic trace amine *N,N*-dimethyltryptamine meets the sigma-1 receptor. Sci. Signal..

[12] Van Waarde A., Rybczynska A.A., Ramakrishnan N.K., Ishiwata K., Elsinga P.H., Dierckx R.A. (2015). Potential applications for sigma receptor ligands in cancer diagnosis and therapy. Biochim. Biophys. Acta.

[13] Bowen W., Hellewell S., McGarry K. (1989). Evidence for a multi-site model of the rat brain sigma receptor. Eur. J. Pharmacol..

[14] Kitaichi K., Chabot J.G., Moebius F.F., Flandorfer A., Glossmann H., Quirion R. (2000). Expression of the purported sigma_1_ (σ_1_) receptor in the mammalian brain and its possible relevance in deficits induced by antagonism of the NMDA receptor complex as revealed using an antisense strategy. J. Chem. Neuroanat..

[15] Skuza G., Rogoz Z. (2006). The synergistic effect of selective sigma receptor agonists and uncompetitive NMDA receptor antagonists in the forced swim test in rats. J. Physiol. Pharmacol..

[16] Yang S., Bhardwaj A., Cheng J., Alkayed N.J., Hurn P.D., Kirsch J.R. (2007). Sigma receptor agonists provide neuroprotection in vitro by preserving bcl-2. Anesth. Analg..

[17] Alon A., Schmidt H.R., Wood M.D., Sahn J.J., Martin S.F., Kruse A.C. (2017). Identification of the gene that codes for the σ2 receptor. Proc. Natl. Acad. Sci. USA.

[18] Myers A.M., Charifson P.S., Owens C.E., Kula N.S., McPhail A.T., Baldessarini R.J., Booth R.G., Wyrick S.D. (1994). Conformational analysis, pharmacophore identification, and comparative molecular field analysis of ligands for the neuromodulatory sigma 3 receptor. J. Med. Chem..

[19] Hayashi T., Su P.T. (2007). Sigma-1 receptor chaperones at the ER-mitochondrion interface regulate Ca(2+) signaling and cell survival. Cell.

[20] Mégalizzi V., Mathieu V., Mijatovic T., Gailly P., Debeir O., De Neve N., Van Damme M., Bontempi G., Haibe-Kains B., Decaestecker C. (2007). Tissue microen- vironment modulates CXCR4 expression and tumor metastasis in Neuroblastoma. Neoplasia.

[21] Aliprantis A.O., Yang R.-B., Mark M.R., Suggett S., Devaux B., Radolf J.D., Klimpel G.R., Godowski P., Zychlinsky A. (1999). Cell activation and apoptosis by bacterial lipoproteins through Toll-like receptor-2. Science.

[22] Kashiwagi H., McDunn E.J., Simon O.P., Goedegebuure S.P., Vangveravong S., Chang K., Hotchkiss S.R., Mach H.R., Hawkins G.W. (2009). Sigma-2 receptor ligands potentiate conventional chemotherapies and improve survival in models of pancreatic adeno- carcinoma. J. Transl. Med..

[23] Ostenfeld M.S., Fehrenbacher N., Hoyer-Hansen M., Thomsen C., Farkas T., Jäättelä M. (2005). Effective tumor cell death by σ-2 receptor ligand Siramesine involves lysosomal leakage and Oxidative Stress. Cancer Res..

[24] Vilner B., John C.S., Bowen W.D. (1995). Sigma-1 and Sigma-2 receptors Are Expressed in a wide variety of human and rodent tumor cell lines. Cancer Res..

[25] Zamora P.O., Moody T.W., John C.S. (1998). Increased binding to sigma sites of *N*-[1-(2-piperidinyl)ethyl)-4-[I-125]-iodobenzamide (I-125-PAB) with onset of tumor cell proliferation. Life Sci..

[26] Spruce B.A., Campbell L.A., McTavish N., Cooper M.A., Appleyard M.V.L., O’Neil M., Howie J., Samson J., Watt S., Murray K. (2004). Small molecule antagonists of the 1 receptor cause selective release of the death program in tumor and self-reliant cells and inhibit tumor growth in vitro and in vivo. Cancer Res..

[27] Colabufo N.A., Berardi F., Contino M., Niso M., Abate C., Perrone R., Tortorella V. (2004). Synthesis, Biological and spectroscopic evaluation of some ligands with intrisinc fluorescent properties. Naunyn Schmiedeberg’s Arch. Pharmacol..

[28] Kim F.J., Maher C.M. (2017). Sigma 1 Pharmacology in the Context of Cancer. Handb. Exp. Pharmacol..

[29] Villard V., Espallergues J., Keller E., Alkam T., Nitta A., Ya-mada K., Nabeshima T., Vamvakides A., Maurice T. (2009). Antiamnesic and neuroprotective effects of the aminotetrahydrofuran derivative ANAVEX1–41 against amyloid beta(25–35)-induced toxicity in mice. Neuropsychopharmacology.

[30] Meunier J., Hayashi T. (2010). Sigma-1 Receptors Regulate Bcl-2 Expression by reactive oxygen species-dependent transcriptional regulation of nuclear factor {kappa}B. J. Pharmacol. Exp. Ther..

[31] Groth-Pedersen L., Ostenfeld M.S., Høyer-Hansen M., Nylandsted J., Jäätelä M. (2007). Vincristine induces dramatic lysosomal changes and sensitizes cancer cells to lysosome-destabilizing siramesine. Cancer Res..

[32] Rui M., Rossi D., Marra A., Paolillo M., Schinelli S., Curti D., Tesei A., Cortesi M., Zamagni A., Laurini E. (2016). Synthesis and biological evaluation of new aryl-alkyl(alkenyl)-4-benzylpiperidines, novel sigma receptor (SR) modulators, as potential anticancer-agents. Eur. J. Med. Chem..

[33] John C.S., Bowen W.D., Varma V.M., McAfee J.G., Moody T.W. (1995). Sigma receptors are expressed in human non-small cell lung carcinoma. Life Sci..

[34] Bem W.T., Thomas G., Mamone J., Homan S.M., Levy B.K., Johnson F.K., Coscia C.J. (1991). Overexpression of sigma receptors in nonneural human tumors. Cancer Res..

[35] Thomas G.E., Szucs M., Mamone J.Y., Bem W.T., Rush M.D., Johnson F.E., Coscia C.J. (1990). Sigma and opioid receptors in human brain tumors. Life Sci..

[36] Zeng C., Rothfuss J., Zhang J., Chu W., Vangveravong S., Tu Z., Pan F., Chang K.C., Hotchkiss R., Mach R.H. (2012). Sigma-2 ligands induce tumour cell death by multiple signalling pathways. Br. J. Cancer.

[37] Mach R., Zeng C., Hawkins G. (2013). The σ2 receptor: A novel protein for the imaging and treatment of cancer. J. Med. Chem..

[38] Su T.P., Su T.C., Nakamura Y., Tsai S.Y. (2016). The sigma-1 receptor as a pluripotent modulator in living systems. Trends Pharmacol. Sci..

[39] Mavlyutov T.A., Guo L.W., Epstein M.L., Ruoho A.E. (2015). Role of the sigma-1 receptor in Amyotrophic Lateral Sclerosis (ALS). J. Pharmacol. Sci..

[40] Alon A., Schmidt H., Zheng S., Kruse A.C., Smith S.B., Su T.P. (2017). Structural perspectives on sigma-1 receptor function. Sigma Receptors: Their Role in Disease and as Therapeutic Targets.

[41] Kekuda R., Prasad P.D., Fei Y.J., Leibach F.H., Ganapathy V. (1996). Cloning and functional expression of the human type 1 sigma receptor (hSigmaR1). Biochem. Biophys. Res. Commun..

[42] Seth P., Ganapathy M.E., Conway S.J., Bridges C.D., Smith S.B., Casellas P., Ganapathy V. (2001). Expression pattern of the type 1 sigma receptor in the brain and identity of critical anionic amino acid residues in the ligand-binding domain of the receptor. Biochim. Biophys. Acta.

[43] Hayashi T., Justinova Z., Hayashi E., Cormaci G., Mori T., Tsai S.-Y., Barnes C., Goldberg S.R., Su T.-P. (2010). Regulation of sigma-1 receptors and endoplasmic reticulum chaperones in the brain of methamphetamine self-administering rats. J. Pharm. Exp. Therap..

[44] Tsai S.-Y., Rothman R.K., Su T.-P. (2012). Insights into the sigma-1 receptor chaperone’s cellular functions: A microarray report. Synapse.

[45] Tsai S.-Y., Hayashi T., Mori T., Su T.-P. (2009). Sigma-1 receptor chaperones and diseases. Cent. Nerv. Syst. Agents Med. Chem..

[46] Soriani O., Vaudry H., Mei Y.A., Roman F., Cazin L. (1998). Sigma ligands stimulate the electrical activity of frog pituitary melanotrope cells through a G-protein-dependent inhibition of potassium conductances. J. Pharmacol. Exp. Ther..

[47] Lupardus P.J., Wilke R.A., Aydar E., Palmer C.P., Chen Y., Ruoho A.E., Jackson M.B. (2000). Membrane-delimited coupling between sigma receptors and K^+^ channels in ratneurohypophysial terminals requires neither G-protein nor ATP. J. Physiol..

[48] Maeno E., Ishizaki Y., Kanaseki T., Hazama A., Okada Y. (2000). Normotonic cell shrinkage because of disordered volume regulation is an early rerequisite to apoptosis. Proc. Natl. Acad. Sci. USA.

[49] Aydar E., Palmer C.P., Klyachko V.A., Jackson M.B. (2002). The sigma receptor as a ligand-regulated auxiliary potassium channel subunit. Neuron.

[50] Fernandez P.C., Frank S.R., Wang L., Schroeder M., Liu S., Greene J., Cocito A., Amati B. (2003). Genomic targets of the human c-Myc protein. Genes Dev..

[51] Das D., Persaud L., Dejoie J., Happy M., Brannigan O., De Jesus D., Sauane M. (2016). Tumor necrosis factor-related apoptosis-inducing ligand (TRAIL) activates caspases in human prostate cancer cells through sigma 1 receptor. Biochem. Biophys. Res. Commun..

[52] Maurice T., Su T.-P. (2009). The pharmacology of sigma-1 receptors. Pharmacol. Ther..

[53] Happy M., Dejoie J., Zajac C.K., Cortez B., Chakraborty K., Aderemi J., Sauane M. (2015). Sigma 1 Receptor antagonist potentiates the anti-cancer effect of p53 by regulating ER stress, ROS production, Bax levels, and caspase-3 activation. Biochem. Biophys. Res. Commun..

[54] Xu C., Zeng W., Chu F., Pan J., Rothfuss M., Zhang F., Tu Z., Zhou D., Zeng D., Vangveravong S. (2011). Identification of the PGRMC1 protein complex as the putative sigma-2 receptor binding site. Nat. Commun..

[55] Huang Y.S., Lu H.L., Zhang L.J., Wu Z. (2014). Sigma-2 receptor ligands and their perspectives in cancer diagnosis and therapy. Med. Res. Rev..

[56] Bowen W.D., Crawford K.W., Huang S., Walker J.W. (2000). Activation of sigma-2 receptors causes changes in ceramide levels in neuronal and non-neuronal cell lines. Soc. Neurosci. Abstr..

[57] Wheeler K.T., Wang L.M., Wallen C.A., Childers S.R., Cline J.M., Keng P.C., Mach R.H. (2000). Sigma-2 receptors as a biomarker of proliferation in solid tumors. Br. J. Cancer.

[58] Toyohara J., Sakata M., Ishiwata K. (2009). Imaging of sigma1 receptors in the human brain using PET and [11C]SA4503. Cent. Nerv. Syst. Agents Med. Chem..

[59] Choi S.-R., Yang B., Plossl K., Chumpradit S., Wey S.-P., Acton P.D., Wheeler K.T., Mach R.H., Kung H.F. (2001). Development of a Tc-99m labeled sigma-2 receptor-specific ligand as a potential breast tumor imaging agent. Nucl. Med. Biol..

[60] John C.S., Gulden M.E., Li J., Bowen W.D., McAfee J.G., Thakur M.L. (1998). Synthesis, in vitro binding, and tissue distribution of radioiodinated 2-[125I]*N*-(*N*-benzylpiperidin-4-yl)-2-iodo benzamide, 2-[125I]BP: A potential sigma receptor marker for human prostate tumors. Nucl. Med. Biol..

[61] Abate C., Perrone R., Berardi F. (2012). Classes of sigma 2 receptor ligands: Structure affinity relationship (SAfiR) studies and antiproliferative activity. Curr. Pharm. Des..

[62] Crawford K.W., Bowen W.D. (2002). Sigma-2 receptor agonists activate a novel apoptotic pathway and potentiate antineoplastic drugs in breast tumor cell lines. Cancer Res..

[63] Mir S.U., Schwarze S.R., Jin L., Zhang J., Friend W., Miriyala D., St Clair D., Craven R.J. (2013). Progesterone receptor membrane component 1/Sigma-2 receptor associates with MAP1LC3B and promotes autophagy. Autophagy.

[64] Bowen W.D., Jin B., Blann E., Vilner B.J., Lyn-Cook B.D. (1997). σ Receptor ligands modulate expression of the multi-drug resistance gene in human and rodent brain tumor cell lines. Proc. Am. Assoc. Cancer Res..

[65] Abate C., Ferorelli S., Contino M., Marottoli R., Colabufo N.A., Perrone R., Berardi F. (2011). Arylamides hybrids of two high-affinity σ2 receptor ligands as tools for the development of PET radiotracers. Eur. J. Med. Chem..

[66] Walker J.M., Bowen W.D., Walker F.O., Matsumoto R.R., De Costa B., Rice K.C. (1990). Sigma receptors: Biology and function. Pharmacol. Rev..

[67] De Costa B.R., He X., Itzhak Y. (1994). Structure-activity relationships and evolution of σ receptor ligands. Sigma Receptors.

[68] Glennon R.A., Ablordeppey S.Y., Ismaiel A.M., El-Ashmawy M.B., Fischer J.B., Burke Howie K. (1994). Structural features important for sigma-1 receptor binding. J. Med. Chem..

[69] Glennon R.A., Smith J.D., Ismaiel A.M., El-Ashmawy M.B., Battaglia G., Fischer J.B. (1991). Identification and exploitation of the sigma-opioid pharmacophore. J. Med. Chem..

[70] Manallack D.T., Wong M.G., Costa M., Andrews P.R., Beart P.M. (1989). Receptor site topographies for phencyclidine-like and sigma drugs: Predictions from quantitative, conformational, electrostatic potential, and radioreceptor analyses. Mol. Pharmacol..

[71] Glennon R.A., Ismaiel A.M., Yousif M., El-Ashmawy M., Herndon J.L., Fischer J.B., Burke Howie K., Server A.C. (1991). Binding of substituted and conformationally restricted derivatives of *N*-(3-phenyl-*n*-propyl)-l-phenyl-2-aminopropanes at sigma receptors. J. Med. Chem..

[72] Glennon R.A., El-Ashmawy M.B., Fischer J.B., Burke Howie K., Ismaiel A.M. (1991). *N*-Substituted 5-phenylpentylamines: A new class of sigma ligands. Med. Chem. Res..

[73] El-Ashmawy M.B., Ablordeppey S.Y., Hassan I., Gad L., Fischer J.B., Burke Howie K., Glennon R.A. (1992). Further investigation of 5-phenylethylamines derivatives as novel sigma receptor ligands. Med. Chem. Res..

[74] Glennon R.A., Yousif M.Y., Ismaiel A.M., El-Ashmawy M.B., Herndon J.L., Fischer J.B., Server A.C., Burke Howie K. (1991). Novel 1-phenylpiperazine and 4-phenylpiperidine derivatives as high affinity sigma ligands. J. Med. Chem..

[75] Ablordeppey S.Y., Issa H., Fischer J.B., Howie B., Glennon R.A. (1993). Synthesis and structure-affinity relationship studies of sigma ligands related to haloperidol. Med. Chem. Res..

[76] Ablordeppey S.Y., Fischer J.B., Law H., Glennon R.A. (2002). Probing the proposed phenyl-A region of the sigma-1 receptor. Bioorg. Med. Chem..

[77] Ablordeppey S.Y., El-Ashmawy M.B., Fischer J.B., Glennon R.A. (1998). A CoMFA investigation of sigma receptor binding affinity: Reexamination of a spurious sigma ligand. Eur. J. Med. Chem..

[78] Itzhak Y. (1994). Sigma Receptors.

[79] Ablordeppey S.Y., Fischer J.B., Glennon R.A. (2000). Is a nitrogen atom an important pharmacophoric element in sigma ligand binding?. Bioorg. Med. Chem..

[80] Ramachandran S., Chu U.B., Mavlyutov T.A., Pal A., Pyne S., Ruoho A.E. (2009). The sigma1 receptor interacts with N-alkyl amines and endogenous sphingolipids. Eur. J. Pharmacol..

[81] Nastasi G., Miceli C., Pittalà V., Modica M.N., Prezzavento O., Romeo G., Rescifina A., Marrazzo A., Amata E. (2017). S2RSLDB: A comprehensive manually curated, internet-accessible database of the sigma-2 receptor selective ligands. J. Cheminform..

[82] Bowen W.D., Bertha C.M., Vilner B.J., Rice K.C. (1995). CB-64D and CB-184: Ligands with high sigma-2 receptor affinity and subtype selectivity. Eur. J. Pharmacol..

[83] Mach R.H., Vangveravong S., Huang Y.S., Yang B., Blair J.B., Wu L. (2003). Synthesis of *N*-substituted 9-azabicyclo[3.3.1]nonan-3α-yl phenylcarbamate analogs as sigma-2 receptor ligands. Med. Chem. Res..

[84] Mach R.H., Wheeler K.T. (2009). Development of molecular probes for imaging sigma-2 receptors in vitro and in vivo. Cent. Nerv. Syst. Agents Med. Chem..

[85] Chu W., Xu J., Zhou D., Zhang F., Jones L.A., Wheeler K.T., Mach R.H. (2009). New *N*-substituted 9-azabicyclo[3.3.1]nonan-3alpha-yl phenylcarbamate analogs as sigma 2 receptor ligands: Synthesis, in vitro characterization, and evaluation as PET imaging and chemosensitization agents. Bioorg. Med. Chem..

[86] Perregaard J., Moltzen E.K., Meier E., Sanchez C. (1995). Sigma ligands with subnanomolar affinity and preference for the sigma 2 binding site. 1. 3-(omega-Aminoalkyl)-1H-indoles. J. Med. Chem..

[87] Moltzen E.K., Perregaard J., Meier E. (1995). Sigma ligands with subnanomolar affinity and preference for the sigma 2 binding site. 2. Spiro-joined benzofuran, isobenzofuran, and benzopyran piperidines. J. Med. Chem..

[88] Zeng C., Vangveravong S., Xu J., Chang K.C., Hotchkiss R.S., Wheeler K.T., Shen D., Zhuang Z.P., Kung H.F., Mach R.H. (2007). Subcellular localization of sigma-2 receptors in breast cancer cells using two-photon and confocal microscopy. Cancer Res..

[89] Mach R.H., Huang Y., Freeman R.A., Wu L., Vangveravong S., Luedtke R.R. (2004). Conformationally-flexible benzamide analogues as dopamine D3 and sigma-2 receptor ligands. Bioorg. Med. Chem. Lett..

[90] Xu R., Lever J.R., Lever S.Z. (2007). Synthesis and in vitro evaluation of tetrahydroisoquinolinylbenzamidesas ligands for sigma receptors. Bioorg. Med. Chem. Lett..

[91] Fan K.H., Lever J.R., Lever S.Z. (2011). Effect of structureal modification in the amine portion of substituted aminobutyl-benzamides as ligands for binding sigma-1 and sigma-2 receptors. Bioorg. Med. Chem..

[92] Hajipour A.R., Guo L.W., Pal A., Mavlyutov T., Ruoho A.E. (2011). Electron-donating para-methoxy converts a benzamide-isoquinoline derivative into a highly sigma-2 receptor selective ligand. Bioorg. Med. Chem..

[93] Berardi F., Abate C., Ferorelli S., Uricchio V., Colabufo N.A., Niso M., Perrone R. (2009). Exploring the importance of piperazine *N*-atoms for σ2 receptor affinity and activity in a series of analogs of 1-cyclohexyl-4-[3-(5-methoxy-1,2,3,4-tetrahydronaphthalen-1-yl)propyl] piperazine (PB28). J. Med. Chem..

[94] Berardi F., Abate C., Ferorelli S., Colabufo N.A., Perrone R. (2009). 1-Cyclohexylpiperazine and 3,3-dimethylpiperidine derivatives as sigma-1 and sigma-2 receptor ligands: A review. Cent. Nerv. Syst. Agents Med. Chem..

[95] Narayanan S., Bhat R., Mesangeau C., Poupaert J.H., McCurdy C.R. (2010). Early development of sigma-receptor ligands. Future Med. Chem..

[96] Akhtar M.J., Ahamed M., Alhadlaq H.A., Alrokayan S.A., Kumar S. (2014). Targeted anticancer therapy: Overexpressed receptors and nanotechnology. Clin. Chim. Acta.

[97] John C.S., Vilner B.J., Gulden M.E., Efange S.M., Langason R.B., Moody T.W., Bowen W.D. (1995). Synthesis and pharmacological characterization of 4-[^125^I]-*N*-(*N*-benzylpiperidin-4-yl)- 4-iodobenzamide: A high affinity sigma receptor ligand for potential imaging of breast cancer. Cancer Res..

[98] Pati M.L., Groza D., Riganti C., Kopecka J., Niso M., Berardi F., Hager S., Heffeter P., Hirai M., Tsugawa H. (2017). Sigma-2 receptor and progesterone receptor membrane component 1 (PGRMC1) are two different proteins: Proofs by fluorescent labeling and binding of sigma-2 receptor ligands to PGRMC1. Pharmacol. Res..

[99] Colabufo N.A., Abate C., Contino M., Inglese C., Niso M., Berardi F., Perrone R. (2008). PB183, a sigma receptor ligand, as a potential PET probe for the imaging of prostate adenocarcinoma. Bioorg. Med. Chem. Lett..

[100] Everaert H., Flamen P., Franken P.R., Verhaeghe W., Bossuyt A. (1997). Sigma-receptor imaging by means of I123-IDAB scintigraphy: Clinical application in melanoma and non-small cell lung cancer. Anticancer Res..

[101] John C.S., Vilner B.J., Geyer B.C., Moody T., Bowen W.D. (1999). Targeting sigma receptor-binding benzamides as in vivo diagnostic and therapeutic agents for human prostate tumors. Cancer Res..

[102] Caveliers V., Everaert H., John C.S., Lahoutte T., Bossuyt A. (2002). Sigma receptor scintigraphy with N-[2-(1′-piperidinyl)ethyl]-3-(123)I-iodo-4-methoxybenzamide of patients with suspected primary breast cancer: First clinical results. J. Nucl. Med..

[103] Michelot J.M., Moreau M.-F.C., Labarre P.G., Madelmont J.-C., Veyre A.J., Papon J.M., Parry D.F., Bonafous J.F., Boire J.-Y.P., Desplanches G.G. (1991). Synthesis and evaluation of new Iodine-125 radiopharmaceuticals as potential tracers for malignant melanoma. J. Nucl. Med..

[104] John C.S., Bowen W.D., Saga T., Kinuya S., Vilner B.J., Baumgold J., Paik C.H., Reba R.C., Neumann R.D., Varma V.M. (1993). A malignant melanoma imaging agent: Synthesis, characterization, in vitro binding and biodistribution of iodine-125-(2-piperidinylaminoethyl)-4-iodobenzamide. J. Nucl. Med..

[105] Banerjee R., Tyagi P., Li S., Huang L. (2004). Anisamide-targeted stealth liposomes: A potent carrier for targeting doxorubicin to human prostate cancer cells. Int. J. Cancer.

[106] Li S.D., Huang L. (2006). Targeted delivery of antisense oligodeoxynucleotide and small interference RNA into lung cancer cells. Mol. Pharm..

[107] Li S.D., Chono S., Huang L. (2008). Efficient oncogene silencing and metastasis inhibition via systemic delivery of siRNA. Mol. Ther..

[108] Bacin F., Michelot J., Bonafous J., Veyre A., Moreau M.-F., Kemeny J.-L., Chossat F., Bekhechi D. (1998). Clinical study of [^123^I] *N*-(2-diethylaminoethyl)-4-iodobenzamide in the diagnosis of primary and metastatic ocular melanoma. Acta Ophthalmol. Scand..

[109] Cachin F., Miot-Noirault E., Gillet B., Isnardi V., Labeille B., Payoux P., Meyer N., Cammilleri S., Gaudy C., Razzouk-Cadet M. (2014). ^123^I-BZA2 as a melanin-targeted radiotracer for the identification of melanoma metastases: Results and perspectives of a multicenter Phase III clinical trial. J. Nucl. Med..

[110] Megalizzi V., Mathieu V., Mijatovic T., Gailly P., Debeir O., De Neve N., Van Damme M., Bontempi G., Haibe-Kains B., Decaestecker C. (2007). 4-IBP, a sigma1 receptor agonist, decreases the migration of human cancer cells, including glioblastoma cells, in vitro and sensitizes them in vitro and in vivo to cytotoxic insults of proapoptotic and proautophagic drugs. Neoplasia.

[111] Megalizzi V., Decaestecker C., Debeir O., Spiegl-Kreinecker S., Berger W., Lefranc F., Kast R.E., Kiss R. (2009). Screening of anti-glioma effects induced by sigma-1 receptor ligands: Potential new use for old anti-psychiatric medicines. Eur. J. Cancer.

[112] Achison M., Boylan M.T., Hupp T.R., Spruce B.A. (2007). HIF-1alpha contributes to tumour-selective killing by the sigma receptor antagonist rimcazole. Oncogene.

[113] Bai T., Wang S., Zhao Y., Zhu R., Wang W., Sun Y. (2017). Haloperidol, a sigma receptor 1 antagonist, promotes ferroptosis in hepatocellular carcinoma cells. Biochem. Biophys. Res. Commun..

[114] Alsharif W., Shen B., Park J.H., Scatliffe S., Chin F., McCurdy C. (2017). Highly Selective sigma-2 receptor PET radioligand [11C]WF197: Radiosynthesis and pilot PET imaging study in mice. J. Nucl. Med..

[115] Hellewell S.B., Bowen W.D. (1990). A sigma-like binding site in rat pheochromocytoma (PC12) cells: Decreased affinity for (+)-benzomorphans and lower molecular weight suggest a different sigma receptor form from that of guinea pig brain. Brain Res..

[116] Hellewell S.B., Bruce A., Feinstein G., Orringer J., Williams W., Bowen W.D. (1994). Rat liver and kidney contain high densities of sigma-1 and sigma-2 receptors: Characterization by ligand binding andphotoaffinity labeling. Eur. J. Pharmacol..

[117] Wang L., Ye J., He Y., Deuther-Conrad W., Zhang J., Zhang X., Cui M., Steinbach J., Huang Y., Brust P. (2017). 18F-Labeled indole-based analogs as highly selective radioligands for imaging sigma-2 receptors in the brain. Bioorg. Med. Chem..

[118] Tu Z., Xu J., Jones L.A., Li S., Dumstorff C., Vangveravong S., Chen D.L., Wheeler K.T., Welch M.J., Mach R.H. (2007). Fluorine-18-labeled benzamide analogues for imaging the sigma-2 receptor status of solid tumors with positron emission tomography. J. Med. Chem..

[119] ClinicalTrials.gov. https://clinicaltrials.gov/ct2/show/NCT03057743.

[120] Vilner B.J., Bowen W.D. (1993). Sigma receptor active neurolepticsare cytotoxic to C6 glioma cells in culture. Eur. J. Pharmacol..

[121] Vilner B.J., de Costa B.R., Bowen W.D. (1995). Cytotoxic effects of sigma ligands: Sigma receptor-mediated alterations in cellular morphology and viability. J. Neurosci..

[122] Spitzer D., Simon P.O., Kashiwagi H., Xu J., Zeng C., Vangveravong S., Zhou D., Chang K., McDunn J.E., Hornick J.R. (2012). Use of multifunctional sigma-2 receptor ligand conjugates to trigger cancer selective cell death signaling. Cancer Res..

[123] Hornick J.R., Xu J., Vangveravong S., Tu Z., Mitchem J.B., Spitzer D., Goedegebuure P., Mach R.H., Hawkins W.G. (2010). The novel sigma-2 receptor ligand SW43 stabilizes pancreas cancer progression in combination with gemcitabine. Mol. Cancer..

[124] Casellas P., Galiegue S., Bourrie B., Ferrini J.B., Jbilo O., Vidal H. (2004). SR31747A: A peripheral σ ligand with potent antitumor activities. Anti Cancer Drugs.

[125] Marrazzo A., Prezzavento O., Pappalardo M.S., Bousquet E., Iadanza M., Pike V.W., Ronsisvalle G. (2002). Synthesis of (+)- and (−)-*cis*-2-[(1-adamantylamino)-methyl]-1-phenyl-cyclopropane derivatives as high affinity probes for σ1 and σ 2 binding sites. IL Farmaco.

[126] Franchini S., Battisti U.M., Prandi A., Tait A., Borsari C., Cichero E., Fossa P., Cilia A., Prezzavento O., Ronsisvalle S. (2016). Scouting new sigma receptor ligands: Synthesis, pharmacological evaluation and molecular modeling of 1,3-dioxolane-based structures and derivatives. Eur. J. Med. Chem..

[127] Yang D., Comeau A., Bowen W.D., Mach R.H., Ross B.D., Hong H., Van Dort M.E. (2017). Design and investigation of a [18F]-labeled benzamide derivative as a high affinity dual sigma receptor subtype radioligand for prostate tumor imaging. Mol. Pharm..

[128] Lever J.R., Litton T.P., Fergason-Cantrell E.A. (2015). Characterization ofpulmonary sigma receptors by radioligand binding. Eur. J. Pharm..

[129] Wanka L., Iqbal K., Schreiner P.R. (2013). The lipophilic bullet hits the targets: Medicinal chemistry of adamantane derivatives. Chem. Rev..

[130] Spilovsk K., Zemek F., Korabecny J., Nepovimova E., Soukup O., Windisch M., Kuca K. (2016). Adamantane—A Lead Structure for Drugs in Clinical Practice. Curr. Med. Chem..

[131] Kornhuber J., Schoppmeyer K., Riederer P. (1993). Affinity of 1-aminoadamantanes for the binding site in post-mortem human frontal cortex. Neurosci. Lett..

[132] Ronsisvalle G., Marrazzo A., Prezzavento O., Pasquinucci L., Falcucci B., Di Toro R., Spampinato S. (2000). Substituted 1-phenyl-2-cyclopropylmethylamines with high affinity and selectivity for sigma sites. Bioorg. Med. Chem..

[133] Bourrie B., Bribes E., De Nys N., Esclangon M., Garcia L., Galiegue S., Lair P., Paul R., Thomas C., Vernieres J.-C. (2002). SSR125329A, a high affinity sigma receptor ligand with potent anti-inflammatory properties. Eur. J. Pharmacol..

[134] Bucolo C., Marrazzo A., Ronsisvalle S., Ronsisvalle G., Cuzzocrea S., Mazzon E., Caputi A., Drago F. (2006). A novel adamantane derivative attenuates retinal ischemia–reperfusion damage in the rat retina through σ1 receptors. Eur. J. Pharmacol..

[135] Banister S.D., Yoo D.T., Wern Chua S., Cui J., Mach R.H., Kassiou M. (2011). *N*-Arylalkyl-2-azaadamantanes as cage-expanded polycarbocyclic sigma (σ) receptor ligands. Bioorg. Med. Chem. Lett..

[136] Riganas S., Papanastasiou I., Foscolos G.B., Tsotinis A., Serin G., Mirjolet J.-F., Dimas K., Kourafalos V.N., Eleutheriades A., Moutsos V.I. (2012). New adamantane phenylalkylamines with σ-receptor binding affinity and anticancer activity, associated with putative antagonism of neuropathic pain. J. Med. Chem..

[137] Riganas S., Papanastasiou I., Foscolos G.B., Tsotinis A., Bourguignon J.J., Serin G., Mirjolet J.F., Dimas K., Kourafalos V.N., Eleutheriades A. (2012). Synthesis, σ1, σ2-receptors binding affinity and antiproliferative action of new C1-substituted adamantanes. Bioorg. Med. Chem..

[138] Riganas S., Papanastasiou I., Foscolos G.B., Tsotinis A., Dimas K., Kourafalos V.N., Eleutheriades A., Moutsos V.I., Khan H., Margarita P. (2012). New adamantane derivatives with sigma affinity and antiproliferative activity. Med. Chem..

[139] Fytas C., Zoidis G., Tsotinis A., Fytas G., Khan M.A., Akhtar S., Rahman K.M., Thurston D.E. (2015). Novel 1-(2-aryl-2-adamantyl)piperazine derivatives with antiproliferative activity. Eur. J. Med. Chem..

[140] Ferle-Vidović A., Kaštelan M., Petrović D., Šuman L., Kaselj M., Škare D., Mlinarić-Majerski K. (1993). Synthesis and biological activity of phencyclidine and its adamantylamine derivatives. Eur. J. Med. Chem..

[141] Papanastasiou I., Riganas S., Foscolos G.B., Tsotinis A., Akhtar S.A., Khan M.A., Rahman K.M., Thurston D.E. (2015). Synthesis and Cytotoxicity of 4-(2-Adamantyl)phenylalkylamines. Lett. Org. Chem..

[142] Koperniku A., Foscolos A.-S., Papanastasiou I., Foscolos G.B., Tsotinis A., Schols D. (2016). 4-(1-Adamantyl)phenylalkylamines with Potential Antiproliferative Activity. Lett. Org. Chem..

[143] Papanastasiou I., Tsotinis A., Kolocouris N., Nikas S.P., Vamvakides A. (2014). New aminoadamantane derivatives with antiproliferative activity. Med. Chem. Res..

